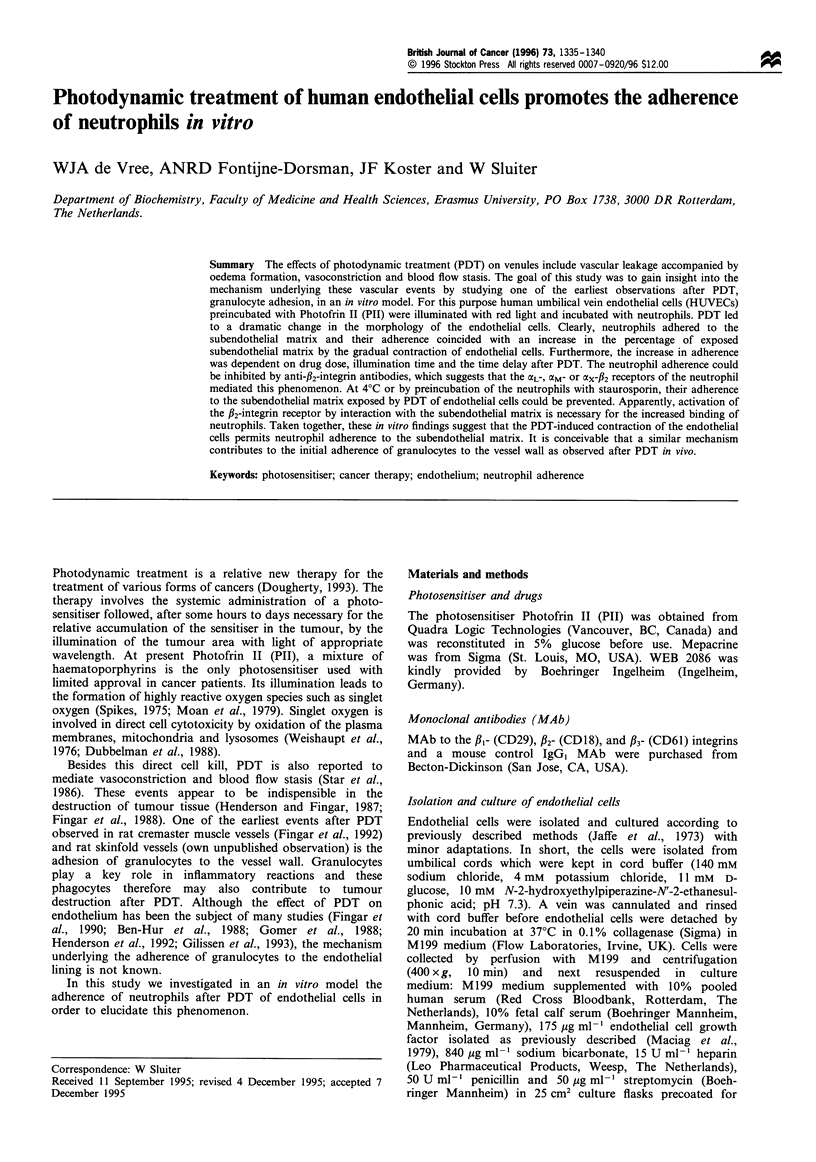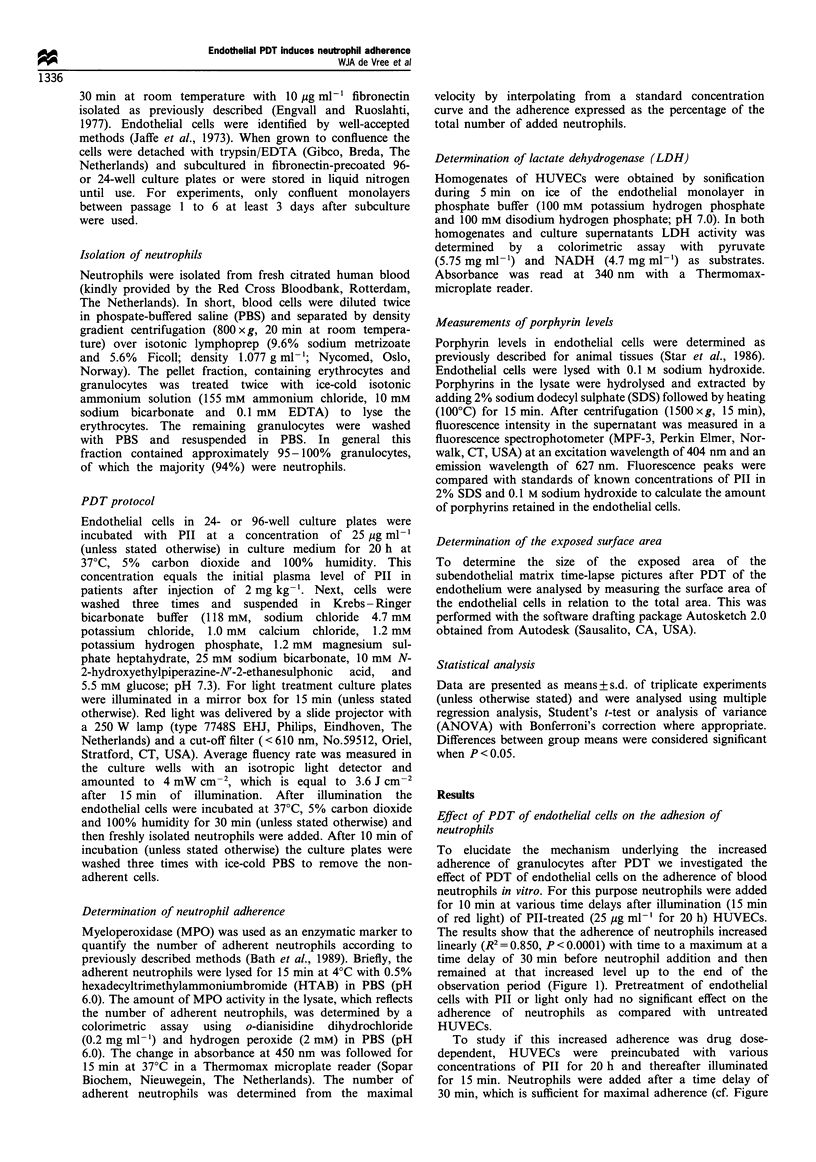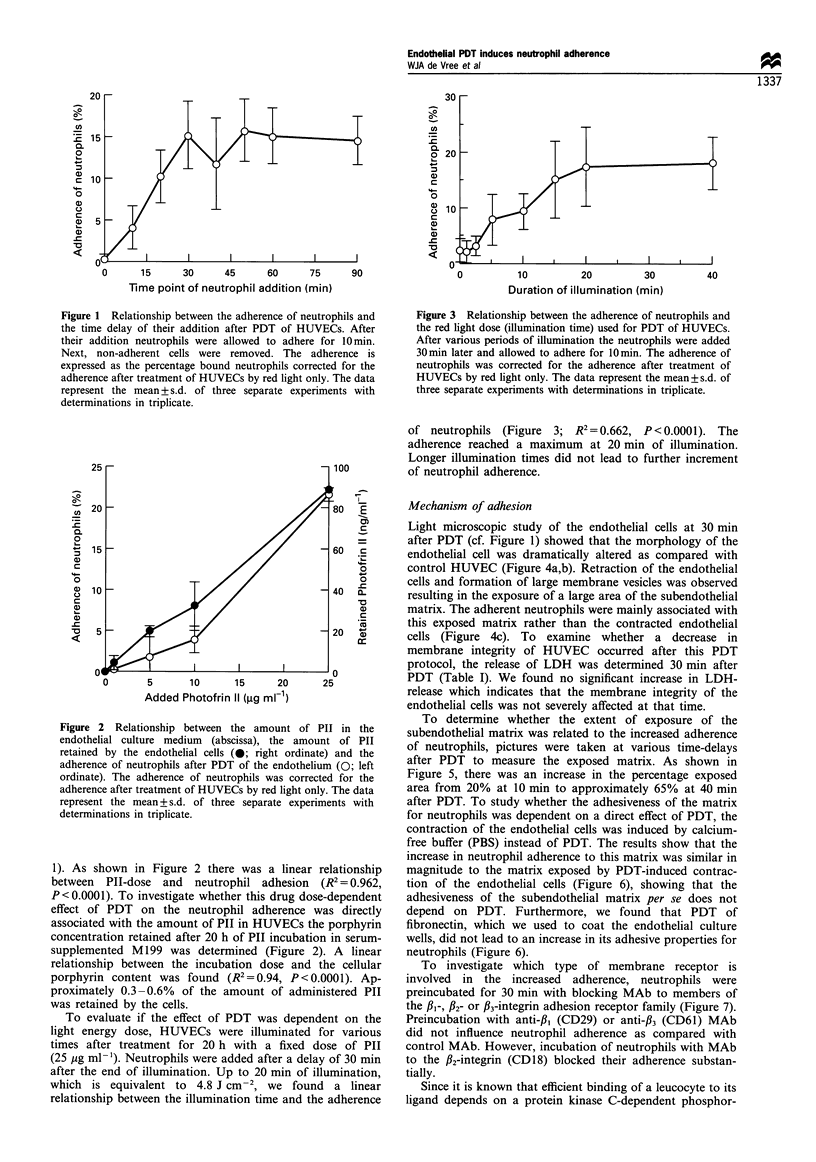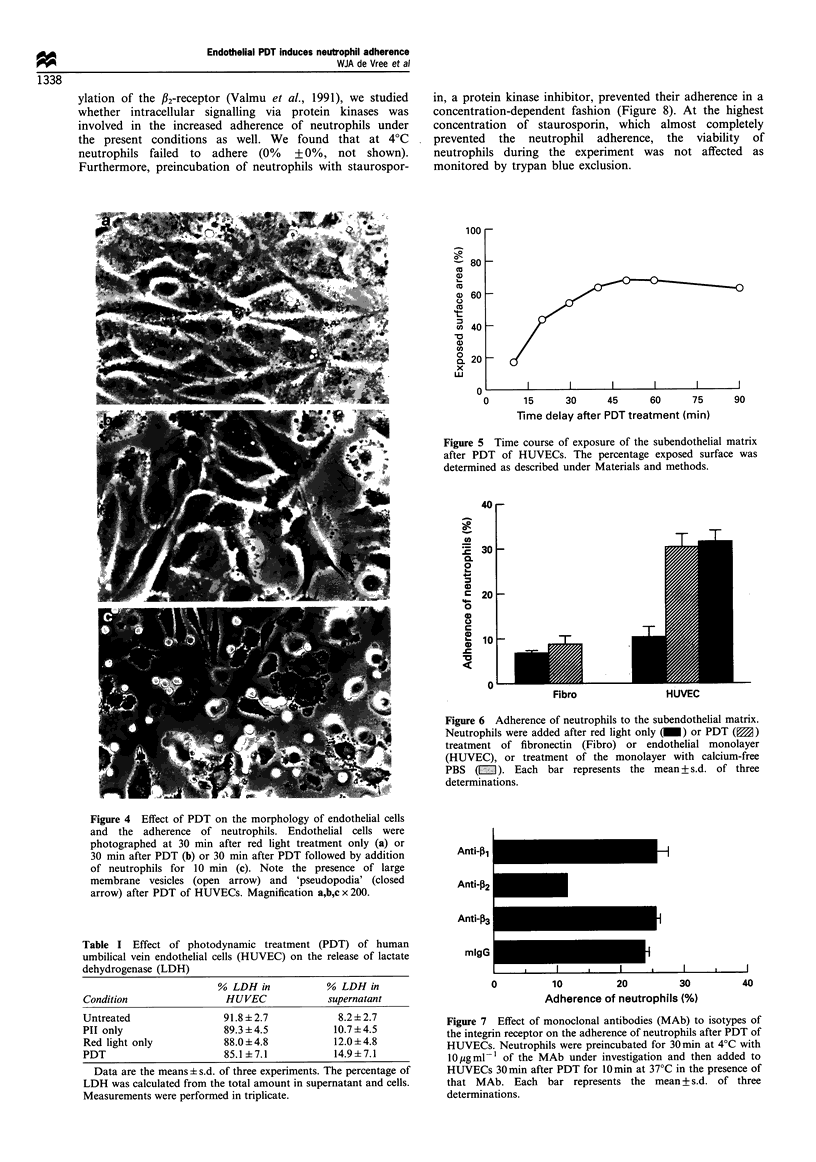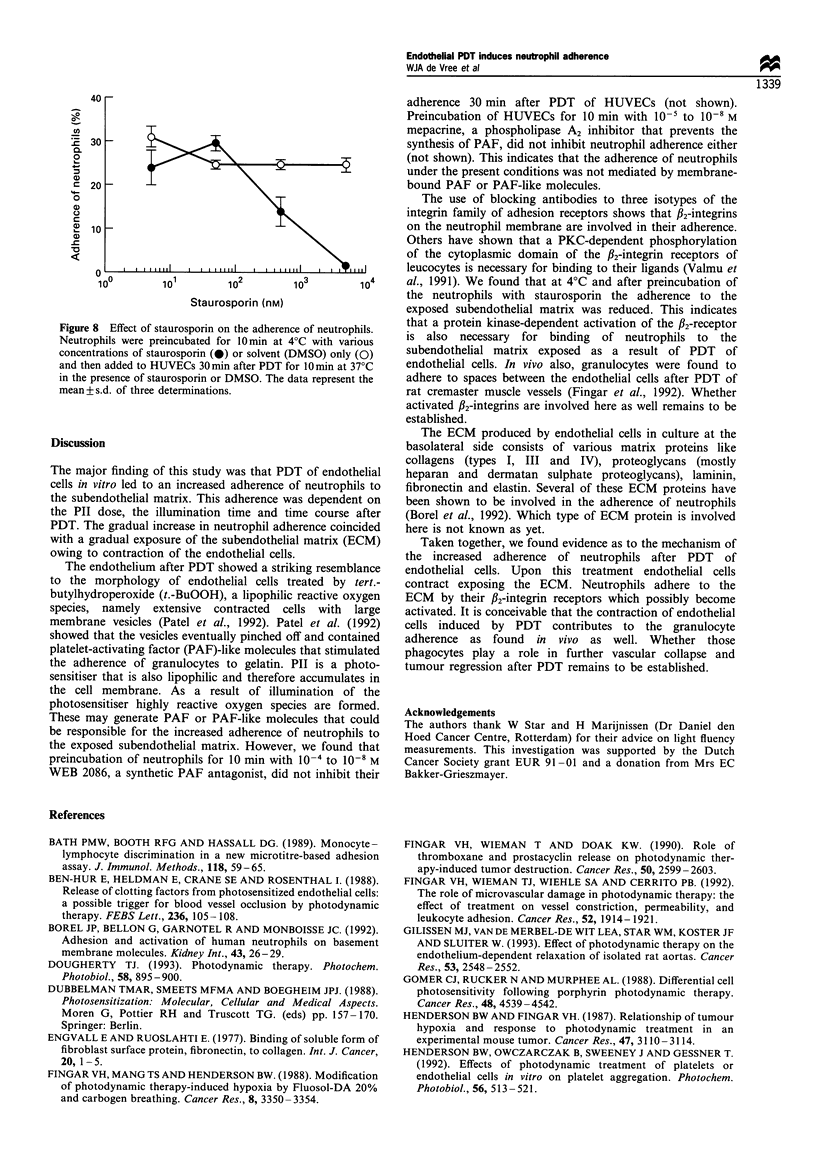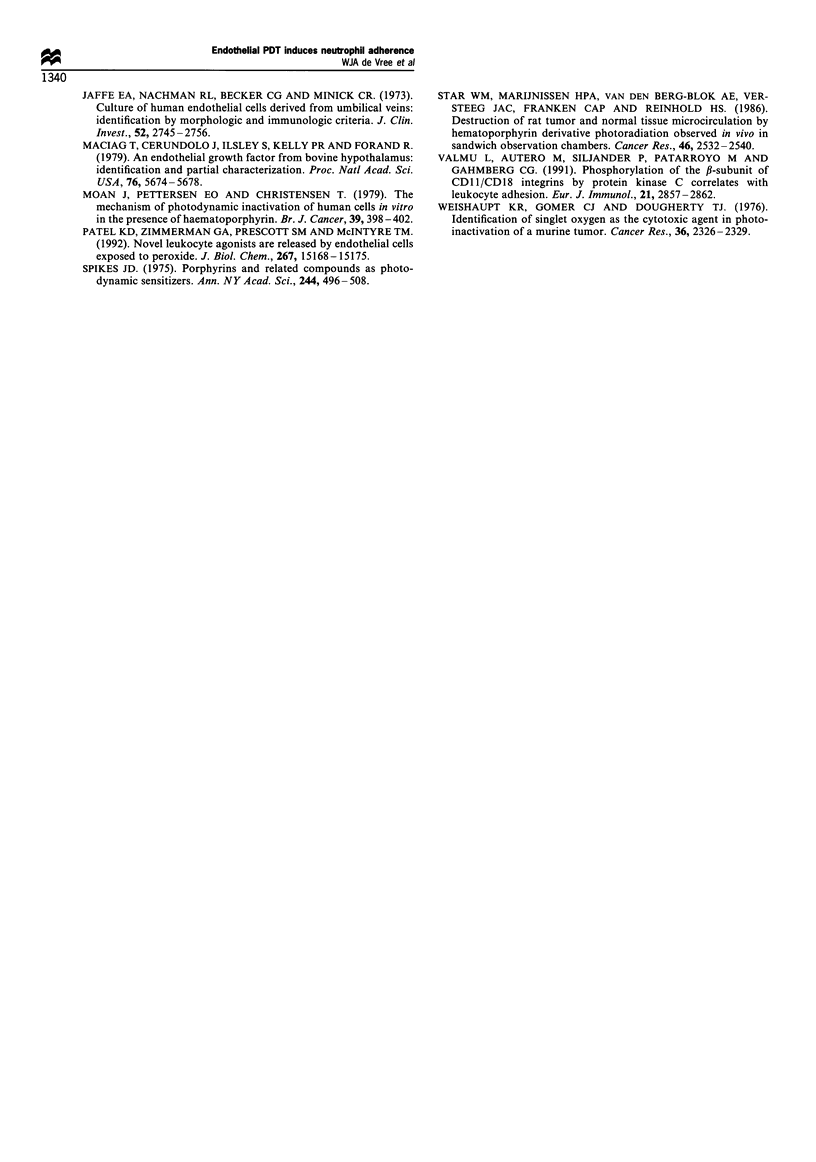# Photodynamic treatment of human endothelial cells promotes the adherence of neutrophils in vitro.

**DOI:** 10.1038/bjc.1996.255

**Published:** 1996-06

**Authors:** W. J. de Vree, A. N. Fontijne-Dorsman, J. F. Koster, W. Sluiter

**Affiliations:** Department of Biochemistry, Faculty of Medicine and Health Sciences, Erasmus University, Rotterdam, The Netherlands.

## Abstract

**Images:**


British Journal of Cancer (1996) 73, 1335-1340
© 1996 Stockton Press All rights reserved 0007-0920/96 $12.00

### Photodynamic treatment of human endothelial cells promotes the adherence of neutrophils *in vitro*

WJA de Vree, ANRD Fontijne-Dorsman, JF Koster and W Sluiter

*Department of Biochemistry, Faculty of Medicine and Health Sciences, Erasmus University, PO Box 1738, 3000 DR Rotterdam, The Netherlands.*

**Summary** The effects of photodynamic treatment (PDT) on venules include vascular leakage accompanied by oedema formation, vasoconstriction and blood flow stasis. The goal of this study was to gain insight into the mechanism underlying these vascular events by studying one of the earliest observations after PDT, granulocyte adhesion, in an *in vitro* model. For this purpose human umbilical vein endothelial cells (HUVECs) preincubated with Photofrin II (PII) were illuminated with red light and incubated with neutrophils. PDT led to a dramatic change in the morphology of the endothelial cells. Clearly, neutrophils adhered to the subendothelial matrix and their adherence coincided with an increase in the percentage of exposed subendothelial matrix by the gradual contraction of endothelial cells. Furthermore, the increase in adherence was dependent on drug dose, illumination time and the time delay after PDT. The neutrophil adherence could be inhibited by anti-$\beta_2$-integrin antibodies, which suggests that the $\alpha_L$-, $\alpha_M$- or $\alpha_X$-$\beta_2$ receptors of the neutrophil mediated this phenomenon. At 4°C or by preincubation of the neutrophils with staurosporin, their adherence to the subendothelial matrix exposed by PDT of endothelial cells could be prevented. Apparently, activation of the $\beta_2$-integrin receptor by interaction with the subendothelial matrix is necessary for the increased binding of neutrophils. Taken together, these *in vitro* findings suggest that the PDT-induced contraction of the endothelial cells permits neutrophil adherence to the subendothelial matrix. It is conceivable that a similar mechanism contributes to the initial adherence of granulocytes to the vessel wall as observed after PDT *in vivo*.

**Keywords:** photosensitiser; cancer therapy; endothelium; neutrophil adherence

Photodynamic treatment is a relative new therapy for the treatment of various forms of cancers (Dougherty, 1993). The therapy involves the systemic administration of a photosensitiser followed, after some hours to days necessary for the relative accumulation of the sensitiser in the tumour, by the illumination of the tumour area with light of appropriate wavelength. At present Photofrin II (PII), a mixture of haematoporphyrins is the only photosensitiser used with limited approval in cancer patients. Its illumination leads to the formation of highly reactive oxygen species such as singlet oxygen (Spikes, 1975; Moan *et al.*, 1979). Singlet oxygen is involved in direct cell cytotoxicity by oxidation of the plasma membranes, mitochondria and lysosomes (Weishaupt *et al.*, 1976; Dubbelman *et al.*, 1988).

Besides this direct cell kill, PDT is also reported to mediate vasoconstriction and blood flow stasis (Star *et al.*, 1986). These events appear to be indispensible in the destruction of tumour tissue (Henderson and Fingar, 1987; Fingar *et al.*, 1988). One of the earliest events after PDT observed in rat cremaster muscle vessels (Fingar *et al.*, 1992) and rat skinfold vessels (own unpublished observation) is the adhesion of granulocytes to the vessel wall. Granulocytes play a key role in inflammatory reactions and these phagocytes therefore may also contribute to tumour destruction after PDT. Although the effect of PDT on endothelium has been the subject of many studies (Fingar *et al.*, 1990; Ben-Hur *et al.*, 1988; Gomer *et al.*, 1988; Henderson *et al.*, 1992; Gilissen *et al.*, 1993), the mechanism underlying the adherence of granulocytes to the endothelial lining is not known.

In this study we investigated in an *in vitro* model the adherence of neutrophils after PDT of endothelial cells in order to elucidate this phenomenon.

Correspondence: W Sluiter
Received 11 September 1995; revised 4 December 1995; accepted 7 December 1995

### Materials and methods

### *Photosensitiser and drugs*

The photosensitiser Photofrin II (PII) was obtained from Quadra Logic Technologies (Vancouver, BC, Canada) and was reconstituted in 5% glucose before use. Mepacrine was from Sigma (St. Louis, MO, USA). WEB 2086 was kindly provided by Boehringer Ingelheim (Ingelheim, Germany).

### *Monoclonal antibodies (MAb)*

MAb to the $\beta_1$- (CD29), $\beta_2$- (CD18), and $\beta_3$- (CD61) integrins and a mouse control $IgG_1$ MAb were purchased from Becton-Dickinson (San Jose, CA, USA).

### *Isolation and culture of endothelial cells*

Endothelial cells were isolated and cultured according to previously described methods (Jaffe *et al.*, 1973) with minor adaptations. In short, the cells were isolated from umbilical cords which were kept in cord buffer (140 mM sodium chloride, 4 mM potassium chloride, 11 mM D-glucose, 10 mM *N*-2-hydroxyethylpiperazine-*N'*-2-ethanesulphonic acid; pH 7.3). A vein was cannulated and rinsed with cord buffer before endothelial cells were detached by 20 min incubation at 37°C in 0.1% collagenase (Sigma) in M199 medium (Flow Laboratories, Irvine, UK). Cells were collected by perfusion with M199 and centrifugation ($400 \times g$, 10 min) and next resuspended in culture medium: M199 medium supplemented with 10% pooled human serum (Red Cross Bloodbank, Rotterdam, The Netherlands), 10% fetal calf serum (Boehringer Mannheim, Mannheim, Germany), 175 $\mu g\ ml^{-1}$ endothelial cell growth factor isolated as previously described (Maciag *et al.*, 1979), 840 $\mu g\ ml^{-1}$ sodium bicarbonate, 15 $U\ ml^{-1}$ heparin (Leo Pharmaceutical Products, Weesp, The Netherlands), 50 $U\ ml^{-1}$ penicillin and 50 $\mu g\ ml^{-1}$ streptomycin (Boehringer Mannheim) in 25 $cm^2$ culture flasks precoated for

30 min at room temperature with 10 $\mu$g ml$^{-1}$ fibronectin isolated as previously described (Engvall and Ruoslahti, 1977). Endothelial cells were identified by well-accepted methods (Jaffe *et al.*, 1973). When grown to confluence the cells were detached with trypsin/EDTA (Gibco, Breda, The Netherlands) and subcultured in fibronectin-precoated 96- or 24-well culture plates or were stored in liquid nitrogen until use. For experiments, only confluent monolayers between passage 1 to 6 at least 3 days after subculture were used.

*Isolation of neutrophils*

Neutrophils were isolated from fresh citrated human blood (kindly provided by the Red Cross Bloodbank, Rotterdam, The Netherlands). In short, blood cells were diluted twice in phospate-buffered saline (PBS) and separated by density gradient centrifugation (800 x $g$, 20 min at room temperature) over isotonic lymphoprep (9.6% sodium metrizoate and 5.6% Ficoll; density 1.077 g ml$^{-1}$; Nycomed, Oslo, Norway). The pellet fraction, containing erythrocytes and granulocytes was treated twice with ice-cold isotonic ammonium solution (155 mM ammonium chloride, 10 mM sodium bicarbonate and 0.1 mM EDTA) to lyse the erythrocytes. The remaining granulocytes were washed with PBS and resuspended in PBS. In general this fraction contained approximately 95–100% granulocytes, of which the majority (94%) were neutrophils.

*PDT protocol*

Endothelial cells in 24- or 96-well culture plates were incubated with PII at a concentration of 25 $\mu$g ml$^{-1}$ (unless stated otherwise) in culture medium for 20 h at 37°C, 5% carbon dioxide and 100% humidity. This concentration equals the initial plasma level of PII in patients after injection of 2 mg kg$^{-1}$. Next, cells were washed three times and suspended in Krebs–Ringer bicarbonate buffer (118 mM, sodium chloride 4.7 mM potassium chloride, 1.0 mM calcium chloride, 1.2 mM potassium hydrogen phosphate, 1.2 mM magnesium sulphate heptahydrate, 25 mM sodium bicarbonate, 10 mM *N*-2-hydroxyethylpiperazine-*N'*-2-ethanesulphonic acid, and 5.5 mM glucose; pH 7.3). For light treatment culture plates were illuminated in a mirror box for 15 min (unless stated otherwise). Red light was delivered by a slide projector with a 250 W lamp (type 7748S EHJ, Philips, Eindhoven, The Netherlands) and a cut-off filter (< 610 nm, No.59512, Oriel, Stratford, CT, USA). Average fluency rate was measured in the culture wells with an isotropic light detector and amounted to 4 mW cm$^{-2}$, which is equal to 3.6 J cm$^{-2}$ after 15 min of illumination. After illumination the endothelial cells were incubated at 37°C, 5% carbon dioxide and 100% humidity for 30 min (unless stated otherwise) and then freshly isolated neutrophils were added. After 10 min of incubation (unless stated otherwise) the culture plates were washed three times with ice-cold PBS to remove the non-adherent cells.

*Determination of neutrophil adherence*

Myeloperoxidase (MPO) was used as an enzymatic marker to quantify the number of adherent neutrophils according to previously described methods (Bath *et al.*, 1989). Briefly, the adherent neutrophils were lysed for 15 min at 4°C with 0.5% hexadecyltrimethylammoniumbromide (HTAB) in PBS (pH 6.0). The amount of MPO activity in the lysate, which reflects the number of adherent neutrophils, was determined by a colorimetric assay using *o*-dianisidine dihydrochloride (0.2 mg ml$^{-1}$) and hydrogen peroxide (2 mM) in PBS (pH 6.0). The change in absorbance at 450 nm was followed for 15 min at 37°C in a Thermomax microplate reader (Sopar Biochem, Nieuwegein, The Netherlands). The number of adherent neutrophils was determined from the maximal velocity by interpolating from a standard concentration curve and the adherence expressed as the percentage of the total number of added neutrophils.

*Determination of lactate dehydrogenase (LDH)*

Homogenates of HUVECs were obtained by sonification during 5 min on ice of the endothelial monolayer in phosphate buffer (100 mM potassium hydrogen phosphate and 100 mM disodium hydrogen phosphate; pH 7.0). In both homogenates and culture supernatants LDH activity was determined by a colorimetric assay with pyruvate (5.75 mg ml$^{-1}$) and NADH (4.7 mg ml$^{-1}$) as substrates. Absorbance was read at 340 nm with a Thermomax-microplate reader.

*Measurements of porphyrin levels*

Porphyrin levels in endothelial cells were determined as previously described for animal tissues (Star *et al.*, 1986). Endothelial cells were lysed with 0.1 M sodium hydroxide. Porphyrins in the lysate were hydrolysed and extracted by adding 2% sodium dodecyl sulphate (SDS) followed by heating (100°C) for 15 min. After centrifugation (1500 x $g$, 15 min), fluorescence intensity in the supernatant was measured in a fluorescence spectrophotometer (MPF-3, Perkin Elmer, Norwalk, CT, USA) at an excitation wavelength of 404 nm and an emission wavelength of 627 nm. Fluorescence peaks were compared with standards of known concentrations of PII in 2% SDS and 0.1 M sodium hydroxide to calculate the amount of porphyrins retained in the endothelial cells.

*Determination of the exposed surface area*

To determine the size of the exposed area of the subendothelial matrix time-lapse pictures after PDT of the endothelium were analysed by measuring the surface area of the endothelial cells in relation to the total area. This was performed with the software drafting package Autosketch 2.0 obtained from Autodesk (Sausalito, CA, USA).

*Statistical analysis*

Data are presented as means ± s.d. of triplicate experiments (unless otherwise stated) and were analysed using multiple regression analysis, Student's *t*-test or analysis of variance (ANOVA) with Bonferroni's correction where appropriate. Differences between group means were considered significant when $P < 0.05$.

### Results

*Effect of PDT of endothelial cells on the adhesion of neutrophils*

To elucidate the mechanism underlying the increased adherence of granulocytes after PDT we investigated the effect of PDT of endothelial cells on the adherence of blood neutrophils *in vitro*. For this purpose neutrophils were added for 10 min at various time delays after illumination (15 min of red light) of PII-treated (25 $\mu$g ml$^{-1}$ for 20 h) HUVECs. The results show that the adherence of neutrophils increased linearly ($R^2 = 0.850$, $P < 0.0001$) with time to a maximum at a time delay of 30 min before neutrophil addition and then remained at that increased level up to the end of the observation period (Figure 1). Pretreatment of endothelial cells with PII or light only had no significant effect on the adherence of neutrophils as compared with untreated HUVECs.

To study if this increased adherence was drug dose-dependent, HUVECs were preincubated with various concentrations of PII for 20 h and thereafter illuminated for 15 min. Neutrophils were added after a time delay of 30 min, which is sufficient for maximal adherence (cf. Figure

**Figure 1** Relationship between the adherence of neutrophils and the time delay of their addition after PDT of HUVECs. After their addition neutrophils were allowed to adhere for 10 min. Next, non-adherent cells were removed. The adherence is expressed as the percentage bound neutrophils corrected for the adherence after treatment of HUVECs by red light only. The data represent the mean ± s.d. of three separate experiments with determinations in triplicate.

**Figure 2** Relationship between the amount of PII in the endothelial culture medium (abscissa), the amount of PII retained by the endothelial cells (●; right ordinate) and the adherence of neutrophils after PDT of the endothelium (○; left ordinate). The adherence of neutrophils was corrected for the adherence after treatment of HUVECs by red light only. The data represent the mean ± s.d. of three separate experiments with determinations in triplicate.

**Figure 3** Relationship between the adherence of neutrophils and the red light dose (illumination time) used for PDT of HUVECs. After various periods of illumination the neutrophils were added 30 min later and allowed to adhere for 10 min. The adherence of neutrophils was corrected for the adherence after treatment of HUVECs by red light only. The data represent the mean ± s.d. of three separate experiments with determinations in triplicate.

1). As shown in Figure 2 there was a linear relationship between PII-dose and neutrophil adhesion ($R^2 = 0.962$, $P < 0.0001$). To investigate whether this drug dose-dependent effect of PDT on the neutrophil adherence was directly associated with the amount of PII in HUVECs the porphyrin concentration retained after 20 h of PII incubation in serum-supplemented M199 was determined (Figure 2). A linear relationship between the incubation dose and the cellular porphyrin content was found ($R^2 = 0.94$, $P < 0.0001$). Approximately 0.3–0.6% of the amount of administered PII was retained by the cells.

To evaluate if the effect of PDT was dependent on the light energy dose, HUVECs were illuminated for various times after treatment for 20 h with a fixed dose of PII (25 μg ml$^{-1}$). Neutrophils were added after a delay of 30 min after the end of illumination. Up to 20 min of illumination, which is equivalent to 4.8 J cm$^{-2}$, we found a linear relationship between the illumination time and the adherence of neutrophils (Figure 3; $R^2 = 0.662$, $P < 0.0001$). The adherence reached a maximum at 20 min of illumination. Longer illumination times did not lead to further increment of neutrophil adherence.

### *Mechanism of adhesion*

Light microscopic study of the endothelial cells at 30 min after PDT (cf. Figure 1) showed that the morphology of the endothelial cell was dramatically altered as compared with control HUVEC (Figure 4a,b). Retraction of the endothelial cells and formation of large membrane vesicles was observed resulting in the exposure of a large area of the subendothelial matrix. The adherent neutrophils were mainly associated with this exposed matrix rather than the contracted endothelial cells (Figure 4c). To examine whether a decrease in membrane integrity of HUVEC occurred after this PDT protocol, the release of LDH was determined 30 min after PDT (Table I). We found no significant increase in LDH-release which indicates that the membrane integrity of the endothelial cells was not severely affected at that time.

To determine whether the extent of exposure of the subendothelial matrix was related to the increased adherence of neutrophils, pictures were taken at various time-delays after PDT to measure the exposed matrix. As shown in Figure 5, there was an increase in the percentage exposed area from 20% at 10 min to approximately 65% at 40 min after PDT. To study whether the adhesiveness of the matrix for neutrophils was dependent on a direct effect of PDT, the contraction of the endothelial cells was induced by calcium-free buffer (PBS) instead of PDT. The results show that the increase in neutrophil adherence to this matrix was similar in magnitude to the matrix exposed by PDT-induced contraction of the endothelial cells (Figure 6), showing that the adhesiveness of the subendothelial matrix *per se* does not depend on PDT. Furthermore, we found that PDT of fibronectin, which we used to coat the endothelial culture wells, did not lead to an increase in its adhesive properties for neutrophils (Figure 6).

To investigate which type of membrane receptor is involved in the increased adherence, neutrophils were preincubated for 30 min with blocking MAb to members of the $\beta_1$-, $\beta_2$- or $\beta_3$-integrin adhesion receptor family (Figure 7). Preincubation with anti-$\beta_1$ (CD29) or anti-$\beta_3$ (CD61) MAb did not influence neutrophil adherence as compared with control MAb. However, incubation of neutrophils with MAb to the $\beta_2$-integrin (CD18) blocked their adherence substantially.

Since it is known that efficient binding of a leucocyte to its ligand depends on a protein kinase C-dependent phosphorylation of the $\beta_2$-receptor (Valmu *et al.*, 1991), we studied whether intracellular signalling via protein kinases was involved in the increased adherence of neutrophils under the present conditions as well. We found that at 4°C neutrophils failed to adhere (0% ±0%, not shown). Furthermore, preincubation of neutrophils with staurosporin, a protein kinase inhibitor, prevented their adherence in a concentration-dependent fashion (Figure 8). At the highest concentration of staurosporin, which almost completely prevented the neutrophil adherence, the viability of neutrophils during the experiment was not affected as monitored by trypan blue exclusion.

**Figure 4** Effect of PDT on the morphology of endothelial cells and the adherence of neutrophils. Endothelial cells were photographed at 30 min after red light treatment only (**a**) or 30 min after PDT (**b**) or 30 min after PDT followed by addition of neutrophils for 10 min (**c**). Note the presence of large membrane vesicles (open arrow) and 'pseudopodia' (closed arrow) after PDT of HUVECs. Magnification **a,b,c** × 200.

**Figure 5** Time course of exposure of the subendothelial matrix after PDT of HUVECs. The percentage exposed surface was determined as described under Materials and methods.

**Figure 6** Adherence of neutrophils to the subendothelial matrix. Neutrophils were added after red light only (■) or PDT (▨) treatment of fibronectin (Fibro) or endothelial monolayer (HUVEC), or treatment of the monolayer with calcium-free PBS (▒). Each bar represents the mean±s.d. of three determinations.

**Figure 7** Effect of monoclonal antibodies (MAb) to isotypes of the integrin receptor on the adherence of neutrophils after PDT of HUVECs. Neutrophils were preincubated for 30 min at 4°C with 10 $\mu g\,ml^{-1}$ of the MAb under investigation and then added to HUVECs 30 min after PDT for 10 min at 37°C in the presence of that MAb. Each bar represents the mean±s.d. of three determinations.

**Table I** Effect of photodynamic treatment (PDT) of human umbilical vein endothelial cells (HUVEC) on the release of lactate dehydrogenase (LDH)

| *Condition* | *% LDH in HUVEC* | *% LDH in supernatant* |
|---|---|---|
| Untreated | 91.8 ± 2.7 | 8.2 ± 2.7 |
| PII only | 89.3 ± 4.5 | 10.7 ± 4.5 |
| Red light only | 88.0 ± 4.8 | 12.0 ± 4.8 |
| PDT | 85.1 ± 7.1 | 14.9 ± 7.1 |

Data are the means ± s.d. of three experiments. The percentage of LDH was calculated from the total amount in supernatant and cells. Measurements were performed in triplicate.

**Figure 8** Effect of staurosporin on the adherence of neutrophils. Neutrophils were preincubated for 10 min at 4°C with various concentrations of staurosporin (●) or solvent (DMSO) only (○) and then added to HUVECs 30 min after PDT for 10 min at 37°C in the presence of staurosporin or DMSO. The data represent the mean ± s.d. of three determinations.

### Discussion

The major finding of this study was that PDT of endothelial cells *in vitro* led to an increased adherence of neutrophils to the subendothelial matrix. This adherence was dependent on the PII dose, the illumination time and time course after PDT. The gradual increase in neutrophil adherence coincided with a gradual exposure of the subendothelial matrix (ECM) owing to contraction of the endothelial cells.

The endothelium after PDT showed a striking resemblance to the morphology of endothelial cells treated by *tert.*-butylhydroperoxide (*t.*-BuOOH), a lipophilic reactive oxygen species, namely extensive contracted cells with large membrane vesicles (Patel *et al.*, 1992). Patel *et al.* (1992) showed that the vesicles eventually pinched off and contained platelet-activating factor (PAF)-like molecules that stimulated the adherence of granulocytes to gelatin. PII is a photosensitiser that is also lipophilic and therefore accumulates in the cell membrane. As a result of illumination of the photosensitiser highly reactive oxygen species are formed. These may generate PAF or PAF-like molecules that could be responsible for the increased adherence of neutrophils to the exposed subendothelial matrix. However, we found that preincubation of neutrophils for 10 min with $10^{-4}$ to $10^{-8}$ M WEB 2086, a synthetic PAF antagonist, did not inhibit their adherence 30 min after PDT of HUVECs (not shown). Preincubation of HUVECs for 10 min with $10^{-5}$ to $10^{-8}$ M mepacrine, a phospholipase $A_2$ inhibitor that prevents the synthesis of PAF, did not inhibit neutrophil adherence either (not shown). This indicates that the adherence of neutrophils under the present conditions was not mediated by membrane-bound PAF or PAF-like molecules.

The use of blocking antibodies to three isotypes of the integrin family of adhesion receptors shows that $\beta_2$-integrins on the neutrophil membrane are involved in their adherence. Others have shown that a PKC-dependent phosphorylation of the cytoplasmic domain of the $\beta_2$-integrin receptors of leucocytes is necessary for binding to their ligands (Valmu *et al.*, 1991). We found that at 4°C and after preincubation of the neutrophils with staurosporin the adherence to the exposed subendothelial matrix was reduced. This indicates that a protein kinase-dependent activation of the $\beta_2$-receptor is also necessary for binding of neutrophils to the subendothelial matrix exposed as a result of PDT of endothelial cells. *In vivo* also, granulocytes were found to adhere to spaces between the endothelial cells after PDT of rat cremaster muscle vessels (Fingar *et al.*, 1992). Whether activated $\beta_2$-integrins are involved here as well remains to be established.

The ECM produced by endothelial cells in culture at the basolateral side consists of various matrix proteins like collagens (types I, III and IV), proteoglycans (mostly heparan and dermatan sulphate proteoglycans), laminin, fibronectin and elastin. Several of these ECM proteins have been shown to be involved in the adherence of neutrophils (Borel *et al.*, 1992). Which type of ECM protein is involved here is not known as yet.

Taken together, we found evidence as to the mechanism of the increased adherence of neutrophils after PDT of endothelial cells. Upon this treatment endothelial cells contract exposing the ECM. Neutrophils adhere to the ECM by their $\beta_2$-integrin receptors which possibly become activated. It is conceivable that the contraction of endothelial cells induced by PDT contributes to the granulocyte adherence as found *in vivo* as well. Whether those phagocytes play a role in further vascular collapse and tumour regression after PDT remains to be established.

**Acknowledgements**
The authors thank W Star and H Marijnissen (Dr Daniel den Hoed Cancer Centre, Rotterdam) for their advice on light fluency measurements. This investigation was supported by the Dutch Cancer Society grant EUR 91-01 and a donation from Mrs EC Bakker-Grieszmayer.

### References

BATH PMW, BOOTH RFG AND HASSALL DG. (1989). Monocyte–lymphocyte discrimination in a new microtitre-based adhesion assay. *J. Immunol. Methods.*, **118,** 59–65.

BEN-HUR E, HELDMAN E, CRANE SE AND ROSENTHAL I. (1988). Release of clotting factors from photosensitized endothelial cells: a possible trigger for blood vessel occlusion by photodynamic therapy. *FEBS Lett.*, **236,** 105–108.

BOREL JP, BELLON G, GARNOTEL R AND MONBOISSE JC. (1992). Adhesion and activation of human neutrophils on basement membrane molecules. *Kidney Int.*, **43,** 26–29.

DOUGHERTY TJ. (1993). Photodynamic therapy. *Photochem. Photobiol.*, **58,** 895–900.

DUBBELMAN TMAR, SMEETS MFMA AND BOEGHEIM JPJ. (1988). *Photosensitization: Molecular, Cellular and Medical Aspects.* Moren G, Pottier RH and Truscott TG. (eds) pp. 157–170. Springer: Berlin.

ENGVALL E AND RUOSLAHTI E. (1977). Binding of soluble form of fibroblast surface protein, fibronectin, to collagen. *Int. J. Cancer,* **20,** 1–5.

FINGAR VH, MANG TS AND HENDERSON BW. (1988). Modification of photodynamic therapy-induced hypoxia by Fluosol-DA 20% and carbogen breathing. *Cancer Res.*, **8,** 3350–3354.

FINGAR VH, WIEMAN T AND DOAK KW. (1990). Role of thromboxane and prostacyclin release on photodynamic therapy-induced tumor destruction. *Cancer Res.*, **50,** 2599–2603.

FINGAR VH, WIEMAN TJ, WIEHLE SA AND CERRITO PB. (1992). The role of microvascular damage in photodynamic therapy: the effect of treatment on vessel constriction, permeability, and leukocyte adhesion. *Cancer Res.*, **52,** 1914–1921.

GILISSEN MJ, VAN DE MERBEL-DE WIT LEA, STAR WM, KOSTER JF AND SLUITER W. (1993). Effect of photodynamic therapy on the endothelium-dependent relaxation of isolated rat aortas. *Cancer Res.*, **53,** 2548–2552.

GOMER CJ, RUCKER N AND MURPHEE AL. (1988). Differential cell photosensitivity following porphyrin photodynamic therapy. *Cancer Res.*, **48,** 4539–4542.

HENDERSON BW AND FINGAR VH. (1987). Relationship of tumour hypoxia and response to photodynamic treatment in an experimental mouse tumor. *Cancer Res.*, **47,** 3110–3114.

HENDERSON BW, OWCZARCZAK B, SWEENEY J AND GESSNER T. (1992). Effects of photodynamic treatment of platelets or endothelial cells *in vitro* on platelet aggregation. *Photochem. Photobiol.*, **56,** 513–521.

JAFFE EA, NACHMAN RL, BECKER CG AND MINICK CR. (1973). Culture of human endothelial cells derived from umbilical veins: identification by morphologic and immunologic criteria. *J. Clin. Invest.*, **52,** 2745–2756.

MACIAG T, CERUNDOLO J, ILSLEY S, KELLY PR AND FORAND R. (1979). An endothelial growth factor from bovine hypothalamus: identification and partial characterization. *Proc. Natl Acad. Sci. USA*, **76,** 5674–5678.

MOAN J, PETTERSEN EO AND CHRISTENSEN T. (1979). The mechanism of photodynamic inactivation of human cells *in vitro* in the presence of haematoporphyrin. *Br. J. Cancer*, **39,** 398–402.

PATEL KD, ZIMMERMAN GA, PRESCOTT SM AND McINTYRE TM. (1992). Novel leukocyte agonists are released by endothelial cells exposed to peroxide. *J. Biol. Chem.*, **267,** 15168–15175.

SPIKES JD. (1975). Porphyrins and related compounds as photodynamic sensitizers. *Ann. NY Acad. Sci.*, **244,** 496–508.

STAR WM, MARIJNISSEN HPA, VAN DEN BERG-BLOK AE, VERSTEEG JAC, FRANKEN CAP AND REINHOLD HS. (1986). Destruction of rat tumor and normal tissue microcirculation by hematoporphyrin derivative photoradiation observed *in vivo* in sandwich observation chambers. *Cancer Res.*, **46,** 2532–2540.

VALMU L, AUTERO M, SILJANDER P, PATARROYO M AND GAHMBERG CG. (1991). Phosphorylation of the $\beta$-subunit of CD11/CD18 integrins by protein kinase C correlates with leukocyte adhesion. *Eur. J. Immunol.*, **21,** 2857–2862.

WEISHAUPT KR, GOMER CJ AND DOUGHERTY TJ. (1976). Identification of singlet oxygen as the cytotoxic agent in photoinactivation of a murine tumor. *Cancer Res.*, **36,** 2326–2329.